# Chrononutrition behaviors during pregnancy: maternal nighttime eating increases the risk of preterm birth

**DOI:** 10.3389/fnut.2025.1699405

**Published:** 2025-11-14

**Authors:** Ameyalli M. Rodríguez-Cano, Berenice Medel-Canchola, Isabel González-Ludlow, Maria Luisa Pizano-Zarate, Guadalupe Estrada-Gutierrez, Otilia Perichart-Perera

**Affiliations:** 1 Nutrition and Bioprogramming Coordination, Instituto Nacional de Perinatología Isidro Espinosa de los Reyes, Mexico City, Mexico; 2 Immunobiochemistry Department, Instituto Nacional de Perinatología Isidro Espinosa de los Reyes, Mexico City, Mexico

**Keywords:** circadian rhythm, prenatal supplementation, maternal diet, neonatal health, perinatal outcomes

## Abstract

**Introduction:**

Maternal nutrition in pregnancy influences birth outcomes and offspring health; chrononutrition behaviors could be potentially linked to perinatal complications such as preterm birth (PTB).

**Aim:**

To evaluate if gestational chrononutrition behaviors influence PTB risk.

**Methods:**

Healthy pregnant women (*n* = 215) from the ongoing OBESO cohort (Mexico City) were studied. Diet (24 h-recall) and sleep-schedule were evaluated in each trimester, obtaining average fasting duration (hours, last–first meal), minutes from waking-up to breakfast (AM-latency), and from dinner to sleeping (PM-latency). Nighttime eating (9:00 pm–5:59 am on three recalls) was registered. Short sleep was defined as <6 h/night. The presence of gestational complications (PE, preeclampsia; GDM, gestational diabetes; GH, gestational hypertension), gestational weight gain (GWG), multivitamin (MV) consumption (low: 0–1 trimesters, moderate: 2 trimesters, high: 3 trimesters) was recorded. Gestational age at resolution was computed and PTB was classified (<37 weeks, first-trimester ultrasound). Logistic regression models were performed to evaluate the association between each chrononutrition behavior and the presence of PTB.

**Results:**

Mean fasting was 11.8 ± 1.2 h; AM, PM latency were 104.4 ± 76.7, 112.3 ± 54.5 min, respectively. Nighttime eating was present in 33% (*n* = 71) of women. Gestational complications prevalence was 16.3% (*n* = 35). Half of women had a high MV-consumption (55.8%, *n* = 120). PTB was present in 8.8% (*n* = 19) of pregnancies. A higher frequency of PTB was observed with nighttime eating (15.5% vs. 5.6%; *p* = 0.16), with low MV-consumption (low: 25%, moderate: 5.6%, high: 7.5%; *p* = 0.011) and with short sleep (63.2% vs. 29.1%; *p* = 0.002). Regression models showed that nighttime eating (OR:5.716, 95%CI:1.724–18.951), low MV-consumption (OR:7.937, 95%CI:1.873–33.639), short sleep (OR:4.551, 95%CI:1.392–14.879) and PE (OR:9.016, 95%CI:1.772–45.881) were positively associated with PTB risk. Other maternal variables in the model (*R*^2^ = 0.318, *p* = 0.010) were not associated (age, obesity, parity, GDM, GWG, energy intake).

**Conclusion:**

Maternal nighttime eating during pregnancy is associated with a higher risk of PTB.

## 1 Introduction

Preterm birth (PTB) remains a major global health challenge, especially in low- and middle-income countries (LMIC), affecting up to 18% of births worldwide and approximately 15 million infants annually. It contributes substantially to neonatal and long-term morbidity and mortality, including neurodevelopmental delay, cerebral palsy, and chronic respiratory conditions, and is associated with an increased risk of adult-onset diseases such as obesity, diabetes, and hypertension. The economic burden is considerable, estimated at over £2.9 billion to the public sector (1).

The precise mechanisms underlying PTB remain unclear. While multiple gestations, infections, and chronic conditions have been identified as potential risk factors, in many cases, no clear etiology is determined. Currently, there are no effective diagnostic tools or early interventions to prevent PTB (1). A growing body of evidence suggests that maternal metabolic health plays a crucial role in pregnancy outcomes. Obesity and inadequate dietary intake contribute to increased oxidative stress, chronic low-grade inflammation, and other alterations in metabolic profiles. This disrupted metabolic environment during pregnancy has been associated with complications such as gestational diabetes mellitus (GDM), preeclampsia (PE), and PTB (2).

Recent studies indicate that maternal dietary patterns may influence PTB risk. A systematic review found that adherence to a healthy diet was associated with a protective effect against PTB (3). Furthermore, the Dietary Inflammatory Index (DII), which quantifies the inflammatory potential of a diet, has been proposed as a predictor of adverse perinatal outcomes. A high DII score, indicating a proinflammatory diet, has been linked to an increased risk of PTB (4). In addition, meta-analyses of RCTs have shown that multiple micronutrient supplementation is also associated with lower risk of PTB, particularly in LMIC (5, 6).

Emerging research in maternal nutrition highlights the significance of chrononutrition—the alignment of dietary intake with circadian rhythms—during pregnancy. This field explores how the timing of food consumption influences metabolic processes, potentially impacting both maternal health and fetal development (7). However, the relationship between chrononutrition and pregnancy outcomes remains scarcely explored (8). A prospective cohort study demonstrated that consuming three regular meals per day was associated with a lower risk of PTB (9). Specifically, maternal nighttime eating—defined as food intake during late evening or nocturnal hours—has been associated with adverse pregnancy outcomes. A recent systematic review highlighted that nighttime eating during pregnancy was linked to a higher risk of impaired glucose metabolism, excessive gestational weight gain, and postpartum weight retention (7). Notably, within this review, only one study reported a significant association between nighttime eating and reduced gestational length, as well as increased odds of preterm birth (10), underscoring the need for further research on this chrononutrition behavior and its potential implications for pregnancy outcomes.

Understanding the impact of nighttime eating and its implications for pregnancy outcomes is crucial for developing dietary recommendations aimed at optimizing maternal and infant health. This study aimed to evaluate whether certain chrononutrition behaviors during pregnancy influence preterm birth risk.

## 2 Materials and methods

### 2.1 Settings

This study is a secondary analysis of the ongoing OBESO (Origen Bioquímico y Epigenético del Sobrepeso y la Obesidad), a prospective perinatal cohort, conducted since 2017 at the Instituto Nacional de Perinatología, Mexico City. The primary aim of the cohort is to evaluate the influence of different maternal factors (including clinical, biochemical, lifestyle, and epigenetics aspects) on infant neurological development and body composition. The OBESO cohort adheres to the ethical standards set forth in the Declaration of Helsinki (11). The study received approval from the Ethics and Research committees (Project No. 3300-11402-01-575-17 and 2024-1-14). Participation was voluntary, with informed written consent obtained from all participating women.

### 2.2 Subjects

For this secondary analysis, participants recruited into the OBESO cohort between 2017 and 2023 were considered. Inclusion criteria for the OBESO cohort were adult women (>18 years old) with a pre-pregnancy body mass index (preBMI) of ≥18.5, carrying a singleton pregnancy (recruited in the first trimester of gestation − 11.0–13.6 weeks). Participants were required to be previously healthy, with no history of conditions such as type 2 diabetes, hypertension, autoimmune diseases, heart, kidney, liver diseases, uncontrolled thyroid disorders, or chronic infections. For this analysis, participants on chronic medications affecting carbohydrate/lipid metabolism, or markers of inflammation/oxidative stress, were excluded, as were those using tobacco, drugs, or alcohol. Additional exclusion criteria included fetal congenital or structural malformations, abnormal fetal karyotype, maternal/fetal infections (e.g., chorioamnionitis), and pregnancy loss. Women missing dietary assessments (fewer than 3 reports), lacking sleep data, working night shifts, or reporting implausible energy intake (<500 or >3,500 kcal/d) were eliminated from this analysis (12).

### 2.3 Prenatal assessment

Maternal assessments included three visits, one during each trimester of pregnancy. During the first visit, procedures included recording self-reported pre-pregnancy weight, measuring height by a trained professional [Lohman’s technique (13), to the nearest 0.1 cm with stadiometer SECA-264, Hamburg, Germany], and computing and classifying preBMI [normal, overweight, obesity; WHO criteria (14)]. Additional clinical and socioeconomic data gathered were parity (nulliparous – no previous childbirth, multiparous – one or more previous childbirths), age (years), socioeconomic status (very low, low, middle/high), occupation (employee/student, housewife), educational level (basic – elementary to middle school; middle – high school or technical education; higher – technical career or bachelor’s/graduate degree).

Each of the three follow-up visits involved measuring weight (to the nearest 0.1 kg; calibrated digital scale, BMB-800, TANITA, Tokyo, Japan) while participants wore light clothing and no shoes. This measurement was then compared to their pre-pregnancy weight, to classify gestational weight gain (GWG) as insufficient, adequate, or excessive, following the recommendations established by the Institute of Medicine (15). Additionally, the follow up visits (second and third trimesters) recorded pregnancy complications (including gestational diabetes mellitus –GDM–, preeclampsia –PE–, gestational hypertension –GH–) and prescribed medications (metformin, insulin), based on self-reports from the participants and verified in the clinical records. Multivitamin (MV) supplementation was documented according to the number of trimesters in which it was consumed: classified as “never/low” if women either did not consume the MV or took it only during one trimester, “moderate” if consumed during two trimesters, and “high” if consumed during all three trimesters. Usual nighttime sleep duration was recorded (only during the first and third trimesters), starting from the moment they fell asleep (not when they went to bed), and short sleep was defined as less than 6 h per night (16). Total physical activity was assessed using the Pregnancy physical activity questionnaire (PPAQ) (17).

### 2.4 Chrononutrition behaviors and dietary assessment

All dietary data were collected through 24-h recalls conducted at each follow-up visit, resulting in a total of three evaluations throughout pregnancy. This process was conducted by a trained and experienced nutrition professional employing a multiple-pass methodology (18). Food replicas and standardized measuring cups and spoons facilitated estimations of portion sizes. Detailed information regarding each eating occasion and the corresponding times was captured during the recalls. Nutrition analysis was obtained from the Food Processor SQL software (version 14.0, ESHA Research, Salem, OR, USA) which provided data on energy (kcal), as well as the grams of protein, carbohydrates, fiber, total fat, monounsaturated, polyunsaturated, saturated fat, and omega-3 fatty acids. The software’s database had been preloaded with standardized local recipes and foods, incorporating information from food labels and Mexican nutritional value tables. For each dietary element, the average was computed from the three dietary assessments.

Chrononutrition behaviors evaluated included:

- Fasting (hours): Calculated as the time interval between the last and first food consumption reported in the 24-h dietary recall, representing the total hours without food. The average from the three trimesters was computed.
- Nighttime eating (yes/no): Participants were classified as nighttime eaters if they reported consuming food outside the daylight period (between 21:00 and 6:00) in all three 24-h dietary recalls. We established these hours considering typical environmental light–dark cycles reported in Mexico City.
- Breakfast latency (minutes): Defined as the time between waking up and consuming the first meal, as recorded in the 24-h dietary recall. The average was calculated using data from the first and third trimesters.
- Dinner latency (minutes): Defined as the time between the last meal consumed, as recorded in the 24-h dietary recall, and bedtime. The average was determined using data from the first and third trimesters.

### 2.5 Preterm birth

Gestational age (in weeks) at the resolution of pregnancy was determined using the first-trimester ultrasound. Preterm birth was defined as delivery occurring before 37 weeks of gestation.

### 2.6 Statistical analysis

To detail the characteristics of the population, descriptive statistics were employed including socioeconomic factors and clinical data, as well as an overview of maternal dietary habits, chrononutrition practices and gestational age at birth preterm birth prevalence. Bivariate analyses were conducted to examine the associations between chrononutrition variables and preterm birth, as well as their relationship with maternal socioeconomic and clinical factors, and with energy and nutrient intake. Depending on data distribution and type, appropriate statistical tests were applied: Pearson or Spearman correlation for continuous variables, Student’s *t*-test or Mann–Whitney *U* test for two-group comparisons, one-way ANOVA or Kruskal–Wallis test for multiple-group comparisons, and Chi-square test for categorical variables.

To identify independent predictors of preterm birth, multivariable logistic regression models were constructed with preterm birth (yes/no) as the dependent variable. Chrononutrition behaviors—including fasting duration, nighttime eating, breakfast latency, and dinner latency—were entered as independent variables. Confounding variables were selected based on prior evidence of their relevance to preterm birth and findings from exploratory analyses. Variables that did not contribute significantly to model performance (i.e., changes in *R*^2^ or *p*-values) were excluded. The final models included maternal age, pre-pregnancy BMI, parity, GWG, total energy intake, MV use, short sleep, and the presence of preeclampsia and GDM. The strength of the models was evaluated using *R*^2^ values, and the normality of residuals was confirmed to satisfy the assumptions of the regression analyses. An OR and a 95% confidence interval (CI) > 1 were deemed statistically significant. All statistical analyses were performed using the SPSS software version 25.0 (IBM, Armonk, NY, USA).

## 3 Results

Of the 458 women enrolled in the OBESO cohort up to 2023, 173 women were eliminated as they did not attend at least three prenatal visits. An additional 69 women were excluded due to incomplete dietary information, and one woman was excluded because of night-shift work. No participants reported implausible energy intakes. In total, 215 pregnant women were included in the final analysis.

### 3.1 Description of pregnant women and chrononutrition behaviors during pregnancy

Maternal characteristics are described in Table 1. Women were mainly housewives, had low socioeconomic status and were nulliparous; most women presented a preBMI of risk and had inadequate (excessive or insufficient) GWG at the end of pregnancy. The majority of women remained without complications (GDM, PE, GH) throughout their pregnancy. Preterm birth was present in 8.8% (*n* = 19) of the sample. Half of the women had a high-MV consumption. Sleep duration ranged from 3.0 to 10.5 h. More than a third of the women had short sleep.

**Table 1:** Maternal characteristics of all women and according to preterm birth.

Maternal characteristics	All (*n* = 215)	Preterm birth		*p*
		No (*n* = 196)	Yes (*n* = 19)	
Age (years)	29.9 ± 5.5	29.9 ± 5.4	30.5 ± 5.8	0.556
Education				
Basic, % (*n*)	22.9 (49)	22.6 (44)	26.3 (5)	0.448
Middle, % (*n*)	39.7 (85)	41.0 (80)	26.3 (5)	
Higher, % (*n*)	37.4 (80)	36.4 (71)	47.4 (9)	
Socioeconomic status				
Very low, % (*n*)	14.0 (30)	13.8 (27)	15.8 (3)	0.758
Low, % (*n*)	50.2 (108)	51.0 (100)	42.1 (8)	
Middle/high, % (*n*)	35.8 (77)	35.2 (69)	42.1 (8)	
Occupation				
Housewife, % (*n*)	64.5 (127)	65.2 (116)	57.9 (11)	0.529
Employee/student, % (*n*)	35.5 (70)	34.8 (62)	42.1 (8)	
Pregestational BMI (kg/m^2^)	26.8 ± 4.8	26.9 ± 4.9	25.5 ± 3.5	0.232
Pregestational BMI status				
Normal, % (*n*)	41.4 (89)	41.3 (81)	42.1 (8)	0.393
Overweight, % (*n*)	36.7 (79)	35.7 (70)	47.4 (9)	
Obesity, % (*n*)	21.9 (47)	23.0 (45)	10.5 (2)	
Parity				
Nulliparous, % (*n*)	59.5 (128)	61.2 (120)	42.1 (8)	0.105
Gestational weight gain				
Insufficient, % (*n*)	34.0 (73)	34.2 (67)	31.6 (6)	0.971
Adequate, % (*n*)	26.0 (56)	26.0 (51)	26.3 (5)	
Excessive, % (*n*)	40.0 (86)	39.8 (78)	42.1 (8)	
Pregnancy complications				
Yes, % (*n*)	16.3 (35)	15.8 (31)	21.1 (4)	0.374
Started metformin/insulin (2nd or 3rd trimester)				
Yes, % (*n*)	10.2 (22)	10.2 (20)	10.5 (2)	0.604
Multivitamin use				
Never/Low, % (*n*)	11.2 (24)	**9.2 (18)**	**31.6 (6)**	**0.011**
Moderate, % (*n*)	33.0 (71)	**34.2 (67)**	**21.1 (4)**	
High, % (*n*)	55.8 (120)	**56.6 (111)**	**47.4 (9)**	
Mean sleep (hours)	6.5 ± 1.4	**6.5 ± 1.4**	**5.8 ± 1.0**	**0.021**
Short sleep (<6 h)				
Yes, % (*n*)	32.1 (69)	**29.1 (57)**	**63.2 (12)**	**0.002**
Total physical activity (MET-hours/week)	190.25 ± 84.7	190.2 ± 83.2	190.5 ± 101.2	0.951

BMI, body mass index; MET, metabolic equivalent of task.

Data presented as mean ± standard deviation or as percentage and number of cases in parenthesis.

Test performed: Student‘s *t*-test/*U* Mann–Whitney test or Chi-squared test. Bold values represent statistically significant values.

Women with preterm birth reported fewer sleep hours. A higher frequency of preterm delivery was observed in women with never/low MV-consumption and with short sleep (Table 1); also, a tendency was identified in women with PE (7.7% vs. 21.1%; *p* = 0.072). Gestational age was not different according to occupation, education, NSE, parity, pre-BMI, GWG, MV-consumption, PE, gestational complications, metformin/insulin use, nighttime eating or short sleep.

From a total of 645 dietary recalls (3 for each woman), the median (25–75°) number of main meals was 2.9 (3.0–3.0), and the time of first and last meal was 9:00 h (8:00–10:00) and 21:00 h (20:30–22:00). Approximately one-third of women (33%, *n* = 71) presented nighttime eating during gestation. The mean breakfast and dinner latency duration were 104.3 ± 76.7 and 112.3 ± 54.5 min, respectively, with a wide distribution (breakfast range: 0 to 540 min, dinner: 0 to 270 min). The mean night fasting duration was 11.8 ± 1.2 h (range 7.9 to 14.7 h).

Nulliparous women showed a shorter breakfast latency (min, 93.9 ± 76.8 vs. 119.5 ± 74.4, *p* = 0.008). They also reported longer sleep duration (h, 6.7 ± 1.4 vs. 6.1 ± 1.3, *p* = 0.011) compared to multiparous women. In contrast, women who identified as housewives had a significantly longer dinner latency than those who were employed or students (min: 120.8 ± 55.4 vs. 102.5 ± 51.1, *p* = 0.027). A higher proportion of women with higher education (basic 36.7%, middle 22.4%, higher 41.3%, *p* = 0.028) and without complications (36.1% vs. 17.1%, *p* = 0.031) reported nighttime eating. A higher frequency of preterm delivery was observed in women with nighttime eating (5.6% vs. 15.5%; *p* = 0.016). Women without pregnancy complications showed less duration of fasting (11.7 ± 1.2 vs. 12.2 ± 1.4, *p* = 0.045).

Maternal age exhibited a negative correlation with hours of fasting (*r* = −0.292, *p* = 0.003) and sleeping (*r* = −0.286, *p* = 0.004). No correlations were identified between chrononutrition behaviors and gestational age. No other differences or correlations were observed in chrononutrition behaviors according to maternal characteristics.

Table 2 describes the mean consumption of energy and macronutrients during pregnancy. Women with nighttime eating presented a higher intake of energy, carbohydrates, total fat, saturated and monounsaturated fat. Fasting hours were negatively correlated with energy (*r* = −0.222, *p* = 0.027), protein (*r* = −0.190, *p* = 0.010), carbohydrates (*r* = −0.224, *p* = 0.002), fiber (*r* = −0.260, *p* < 0.001), total fat (*r* = −0.164, *p* = 0.027), saturated (*r* = −0.162, *p* = 0.029) and monounsaturated (*r* = −0.158, *p* < 0.033). Breakfast latency showed negative correlations with protein (*r* = −0.166, *p* = 0.029) and fiber (*r* = −0.177, *p* = 0.019) consumption. No differences in energy or nutrient intake were identified between women who had preterm birth and those who did not.

**Table 2:** Average energy and nutrient consumption during pregnancy according to nighttime eating and preterm birth.

Nutrient	All (*n* = 215)	Preterm birth			Nighttime eating		
		No (*n* = 196)	Yes (*n* = 19)	*p*	No (*n* = 144)	Yes (*n* = 71)	*p*
Energy (kcal/d)	2111.3 ± 535.9	2115.8 ± 542.4	2048.1 ± 448.3	0.674	**2048.1 ± 529.3**	**2250.0 ± 528.7**	**0.018**
Protein (g/d)	87.5 ± 24.5	88.2 ± 24.9	77.8 ± 14.8	0.225	85.9 ± 24.7	90.9 ± 24.0	0.217
Carbohydrates (g/d)	274.2 ± 71.3	273.6 ± 71.2	282.6 ± 75.3	0.803	**267.7 ± 70.0**	**288.6 ± 72.8**	**0.022**
Fiber (g/d)	26.4 ± 10.2	26.5 ± 10.5	25.2 ± 6.3	0.742	26.2 ± 9.26	26.8 ± 12.3	0.875
Fat (g/d)	75.7 ± 25.8	76.1 ± 26.2	70.9 ± 19.2	0.759	**72.5 ± 25.6**	**82.7 ± 25.1**	**0.006**
Saturated fat (g/d)	21.0 ± 8.3	21.1 ± 8.4	19.6 ± 6.1	0.955	**20.1 ± 8.4**	**22.9 ± 7.8**	**0.012**
Monounsaturated fat (g/d)	24.2 ± 9.0	24.3 ± 9.1	19.6 ± 6.1	0.878	**23.1 ± 9.0**	**26.4 ± 8.5**	**0.014**
Polyunsaturated fat (g/d)	15.2 ± 6.7	15.2 ± 6.8	15.4 ± 6.2	0.856	14.8 ± 6.6	16.1 ± 6.9	0.194

Data presented as mean ± standard deviation.

Test performed: Student’s *t*-test/U Mann–Whitney test. Bold values represent statistically significant values.

### 3.2 Chrononutrition behaviors and their association with preterm birth

According to the logistic regression models, nighttime eating was the only chrononutrition behavior associated with preterm birth. The strongest model (*R*^2^ = 0.318, *p* = 0.001) showed that nighttime eating, developing preeclampsia, never/low MV-consumption, and sleep hours were positively associated with preterm birth risk (Table 3).

**Table 3:** Multivariable analysis of nighttime eating and maternal factors in relation to preterm birth risk.

Predictive variables	*B*	Standard error	OR	95% CI		*p*
				Lower	Higher	
Preeclampsia (yes)	2.199	0.830	9.016	1.772	45.881	0.008
Multivitamin intake (never/low)	2.072	0.737	7.937	1.873	33.639	0.005
Short sleep (yes)	1.515	0.604	4.551	1.392	14.879	0.012
Nighttime eating (yes)	1.743	0.612	5.716	1.724	18.951	0.004
Constant	−1.671	2.751	0.188			0.544

Multiple logistic regression model. Adjusted by maternal age, pregestational body mass index, parity, gestational diabetes, gestational weight gain, and total energy intake during pregnancy.

Reference group for categorical variables: preeclampsia-no, multivitamin intake −3 trimesters, short sleep-no, nighttime eating-no.

## 4 Discussion

In this study, we explored the associations between maternal chrononutrition behaviors during pregnancy and the risk of preterm birth (PTB). Our findings indicate that nighttime eating (between 9:00 pm and 5:59 am) is positively associated with PTB risk.

This finding aligns with emerging evidence that meal timing, particularly late or nocturnal intake, may negatively affect pregnancy outcomes (7). To date, only one other study has reported a similar association: Loy et al. (10) found that night eating during pregnancy was linked to a 0.45-week shortening of gestational length and 2.19-fold increased odds of PTB. The mechanisms underlying this association likely involve circadian misalignment, metabolic disruption, and altered inflammatory pathways. The uterus and the placenta utilize circadian rhythm to carry out certain physiological functions, e.g., hormone release, parturition, and immune function, and entrain the fetus’ circadian rhythm. The development of the embryo, uterine implantation, placentation, and delivery may be regulated by the clock molecular machinery in the circadian rhythm (19). Food intake during the biological night—when melatonin is elevated, and metabolic activity is reduced—can lead to desynchronization between central and peripheral clocks (20, 21). Melatonin and cortisol, two key hormones with distinct circadian patterns, play central roles in this context.

Melatonin peaks between 2:00 and 4:00 and regulates placental function, antioxidant defenses, and immune homeostasis during gestation (19, 22). Teoh et al. (20) reported that pregnant women with higher energy and macronutrient intake after 19:00 had altered melatonin concentrations, further supporting the link between late eating and endocrine disruption. Through its actions on transcription factors such as NF-κB, Nrf2, and HIF-1, melatonin reduces placental oxidative stress and inflammatory signaling (23). The impairment of the circadian rhythm during pregnancy compromises melatonin production (19). Disruption of melatonin rhythms, often seen in pregnancies complicated by placental insufficiency, has been associated with adverse outcomes including preeclampsia, fetal growth restriction, PTB, miscarriage, and low birthweight (24). Impaired melatonin signaling may also contribute to offspring neurodevelopmental and metabolic disorders (22, 23).

Cortisol, a glucocorticoid hormone with a well-defined diurnal rhythm, is typically lowest at night, peaks in the early morning, and progressively declines throughout the day. It plays essential roles in immune regulation, inflammatory control, and metabolism (25). Nighttime intake may stimulate excess nocturnal cortisol secretion, activating the maternal HPA axis and promoting inflammatory pathways that contribute to PTB—though further evidence is needed to confirm this mechanism (26–28). A small, randomized crossover trial comparing late (22:00) vs. routine (18:00) dinners in healthy non-pregnant adults showed that late meals led to higher nocturnal cortisol levels, impaired glucose tolerance, and reduced lipid mobilization (29). In pregnancy, altered cortisol rhythms have been linked to adverse outcomes: a systematic review reported that elevated maternal cortisol levels were associated with intrauterine growth restriction, shortened gestation, low birth weight, impaired infant neurodevelopment, and reduced abundance of potentially beneficial gut microbiota (25).

In our cohort, approximately one-third of pregnant women reported engaging in nighttime eating, which was associated with significantly higher intake of total energy, carbohydrates, and fats—particularly saturated and monounsaturated fat—compared to those without this behavior. This prevalence is consistent with previous reports in pregnant populations (15%–45%), though wide variation exists due to inconsistent definitions and assessment methods (21). Other studies have similarly observed that pregnant night-eaters tend to consume more fat, often alongside lower nutrient density (e.g., reduced calcium and iron intake) (30, 31). Determinants of nighttime eating extend beyond pregnancy-specific physiology and include behavioral, social, and situational factors such as evening hunger, limited time, long work hours, family dynamics, shared mealtimes, stress, boredom, and habitual eating patterns (32). Pregnancy-related symptoms like insomnia or nausea may exacerbate these tendencies. Circadian disruption, such as nighttime eating, has been associated with hormonal and neurobiological alterations that drive overeating and reward-seeking behavior. Misaligned melatonin and cortisol rhythms impair appetite regulation, blunt satiety, and increase cravings for energy-dense foods. Elevated evening cortisol promotes stress-related eating, while reduced nocturnal melatonin weakens metabolic control. Additionally, circadian misalignment and sleep loss disrupt dopaminergic pathways, lowering prefrontal inhibitory control and increasing mesolimbic reward activity—fostering impulsive, hedonic eating tendencies (33, 34).

We observed no association between maternal fasting duration or breakfast/dinner latency with PTB risk in our cohort. Although prolonged fasting is generally discouraged during pregnancy, current evidence remains inconclusive. A recent umbrella review reported no evidence of an increase in PTB risk or reduced gestational length associated with Ramadan fasting during pregnancy (review of 10 studies) (35). To our knowledge, time-restricted feeding (TRF) (extending the nocturnal fasting interval) has not been studied in human pregnancies, nor has the breakfast or dinner latency. However, animal models showed that in obese pregnant rats, TRF reduced high-fat diet–induced placental apoptosis and inflammation, thereby minimizing cellular stress and preserving autophagic function (36). These findings highlight the need for further research to assess the safety, feasibility, and potential benefits of chrononutrition-based interventions during pregnancy.

In addition to nighttime eating, in our cohort, the lack of multivitamin supplementation or low frequency of use was linked to an increase in PTB risk. Various meta-analyses indicate that multiple micronutrient supplementation modestly reduces preterm birth rates, especially in nutritionally vulnerable populations. A 2019 Cochrane review of >140,000 women in mostly LMIC trials found that daily multiple-micronutrient supplementation (vs iron–folic acid) resulted in a slight overall reduction in PTB (RR 0.95, 95%CI 0.90 to 1.01; moderate-quality evidence) and a marked decrease in very preterm deliveries (RR 0.81, 95%CI 0.71 to 0.93) (6). Likewise, individual-patient pooled analyses of RCTs, including women from LMIC, showed an overall reduction in PTB risk with larger PTB risk reductions in high-risk subgroups – for example, underweight women (BMI < 18.5) experienced 16% lower PTB risk on multiple-micronutrient supplementation and early initiation of supplements (<20 weeks) yielded an 11% risk drop (5). These findings have been echoed in recent systematic reviews of LMIC cohorts, including pregnant adolescents, which report similar PTB reductions with multivitamin use compared to standard care (37).

An emerging body of epidemiological evidence suggests that maternal sleep duration is associated with the risk of PTB. In our analysis, less than 6 h of sleep emerged as a relevant predictor of PTB. Several observational studies and meta-analyses have consistently shown that pregnant women who sleep fewer hours per night face higher odds of PTB. A 2020 meta-analysis reported that women with the shortest sleep duration had approximately a 20% higher risk of PTB compared to those with the longest sleep, while poor sleep quality was associated with an even greater increase in risk (~50%) (38). More recent evidence from a dose–response meta-analysis has confirmed these associations and even suggested a non-linear (U-shaped) relationship, wherein both very short and very long sleep durations are associated with elevated PTB risk (39). However, not all studies have found consistent associations; for instance, a large Japanese cohort reported no significant link between nightly sleep duration and PTB—potentially explained by a lower baseline PTB rate in that population (40).

Our multivariate analysis also identified preeclampsia as an independent risk factor for preterm birth. Globally, preeclampsia accounts for an estimated 15%–20% of all preterm births, primarily due to the need for medically indicated early delivery, which remains the only definitive treatment in severe cases (41). The risk is particularly elevated when preeclampsia develops early in pregnancy, with large cohort studies reporting up to an eightfold increase in preterm birth for cases diagnosed before 28 weeks (42). Since our model included both spontaneous and medically indicated preterm births, it is likely that many women with preeclampsia underwent planned early delivery (often by scheduled cesarean section at early-term gestation) to prevent severe maternal or fetal complications. Previous studies have emphasized that medically indicated and spontaneous preterm births arise from different pathophysiological pathways and analyzing them together can mask certain risk relationships (43). Consistent with prior findings, we found no significant associations between maternal age, obesity, parity, GDM, or GWG and preterm birth risk in our cohort (44).

One of the key strengths of this study is its foundation in the OBESO cohort, a well-defined prospective perinatal cohort with high-quality, standardized data collection protocols. This robust design enabled a comprehensive assessment of maternal chrononutrition behaviors across pregnancy. We explored eating habits at three distinct time points, allowing us to capture longitudinal patterns and enhance the reliability of exposure classification. The dietary assessment using multiple-pass methodology aids in memory recall, captures detail and reduces omissions. The precise determination of gestational age via first-trimester ultrasound further improved the accuracy of preterm birth classification. In addition, our analytical models adjusted for several important confounders, enhancing the validity of the observed associations.

At the same time, several limitations should be considered when interpreting the results. As an observational study, our findings are subject to residual confounding and cannot establish causality, even with rigorous adjustment for known variables. The operational definition of nighttime eating—intake occurring outside daylight hours based on three recall days—may not fully capture habitual dietary behaviors, which may have different metabolic implications. The inability to differentiate between spontaneous and medically indicated preterm births may also obscure distinctions in underlying causes. Considering dietary intake and sleep duration were self-reported, some degree of reporting bias is inevitable. Sleep data were collected at only two time points, limiting our capacity to track changes or variability in sleep patterns throughout pregnancy. Additionally, the inability to characterize more specific aspects of meal timing and differences between weekday and weekend eating routines may have limited the accuracy of chrononutrition pattern assessment. Also, the relatively small number of preterm births may have reduced statistical power of our analysis. Our study population, drawn from a specialized tertiary care perinatal center, may not reflect the full diversity of pregnant populations.

Importantly, nighttime eating does not occur in isolation—it often coexists with other interrelated aspects of eating behavior such as the distribution of energy across the day, meal frequency and regularity, and fasting intervals. These components are intricately connected and may jointly influence maternal metabolism and pregnancy outcomes. As such, future studies should adopt a broader chrononutrition framework that captures not only what and how much women eat during pregnancy, but also when, why, and under what circumstances they eat. Incorporating objective assessment tools such as actigraphy or continuous glucose monitoring may help address existing measurement limitations and offer deeper insights. These results contribute to the growing evidence that the timing of maternal nutrition is a critical, potentially modifiable factor in optimizing pregnancy. Our findings underscore the significance of maternal nighttime eating as a modifiable behavioral factor that may be associated with an increased risk of preterm birth. This relationship likely operates through multiple interconnected pathways, including circadian misalignment, hormonal disruptions, metabolic dysfunction, oxidative stress, and inflammation. These results highlight the need to move beyond traditional dietary recommendations focused solely on energy and nutrients, by also considering when and how food is consumed. Integrating chrononutrition principles into prenatal counseling, may offer a novel, low-cost strategy to support healthier pregnancies. Future research should further explore the effectiveness and feasibility of such interventions across diverse populations. Ultimately, incorporating both timing and quality of maternal nutrition into prenatal care can contribute meaningfully to reducing preterm birth risk and improving maternal and neonatal outcomes.

## 5 Conclusion

One third of women showed nighttime eating behavior during pregnancy, and the average nocturnal fasting duration was 11 h. Breakfast and dinner latencies showed wide variability. Short sleep duration (<6 h) was also present in one-third of women. Nighttime eating was associated with a higher risk of preterm birth, while other factors linked to prematurity included short sleep duration, low use of prenatal multivitamins, and preeclampsia. Assessing chrononutrition behaviors during pregnancy may represent a valuable opportunity to identify at-risk women and develop targeted strategies to improve perinatal outcomes.

## Data Availability

The raw data supporting the conclusions of this article will be made available by the authors, without undue reservation.
